# Information needs in breast reconstruction after mastectomy: a qualitative analysis of free-text responses from 2077 women

**DOI:** 10.1007/s10549-023-07240-3

**Published:** 2024-02-01

**Authors:** Kim Wuyts, Vicki Durston, Lisa Morstyn, Sam Mills, Victoria White

**Affiliations:** 1 School of Psychology, Faculty of Health, Deakin University, 1 Gheringhap Street, 3220 Geelong, VIC Australia; 2 Breast Cancer Network Australia, Camberwell, VIC Australia

**Keywords:** Breast reconstruction, Information needs, Women’s experiences, Mastectomy, Breast cancer, Qualitative analyses

## Abstract

**Background:**

For many, breast reconstruction following mastectomy (BR) forms an integral part of breast cancer survivorship. For those considering BR, provision of information is essential to allow informed decisions. Using free-text responses from a survey of breast cancer survivors, this study aims to understand current gaps in information regarding BR.

**Method:**

At the end of an online survey assessing BR experiences, participants were asked the open-ended question: “Thinking about women who may experience BR in the future, is there anything you think needs to change so that they have a better experience?”. Responses were analysed to identify common themes.

**Results:**

3384 people completed the survey with 2,077 (61%) responding to the open-ended question. Three themes were identified: (1) content of information, (2) managing expectations, and (3) information sources, each associated with multiple subthemes. Information wanted in theme (1) covered a range of topics including BR options, risks, recovery and ‘going flat.’ Information on BR’s psychological impact was also needed, with comments indicating many were not prepared for this. Theme (2) stressed the importance of realistic information about BR outcomes and processes to reduce discrepancies between expectations and experiences. In theme (3), peer insights and photos were important sources of realistic information.

**Conclusion:**

Multiple gaps exist in BR-related information available to women. BR information needs to be comprehensive, realistic, and provided at the right time to allow informed decision-making. Developing strategies to strengthen existing information provision as well as new resources to fill information gaps might enhance BR experiences.

## Introduction

In 2022, over 20,000 Australian women were diagnosed with breast cancer [1], with approximately 40% treated by mastectomy [2]. Evidence suggests breast reconstruction (BR) following mastectomy is oncologically safe [3, 4] and associated with improved quality of life, body image, sexuality, and emotional well-being [5–8]. However, the rate of BR after mastectomy in Australia (29% in 2019) [9] is low compared to the USA (43%) [10]. Since the passing of the Breast Cancer Patient Education Act of 2015, federal laws in the USA have required that women considering treatment by mastectomy receive information about BR options [11, 12]. No such mandate exists in Australia, and although Australian clinical practice guidelines recommend that all women undergoing mastectomy for breast cancer are fully informed about BR options before their mastectomy [13], the low BR rate may suggest many women are not receiving adequate information.

A breast cancer diagnosis is associated with high psychological distress [14]. Yet women need to make numerous treatment decisions in a short timeframe, including whether to undergo reconstruction. Women considering BR must choose between immediate BR (IBR) or delayed BR (DBR), implants or autologous tissue flap reconstruction [15] treatment location (public or private hospital), and type of surgeon (breast or plastic surgeon). Research focusing on information needs has identified significant gaps in the type and amount of information provided, including the option of BR, the possibility of having immediate reconstruction, and the different BR procedures [16, 17]. A 2015 survey of 501 Australian women who had undergone a mastectomy found 39% did not have the option to have IBR due to a lack of discussion about BR prior to mastectomy [18]. A US study assessing BR knowledge and decision quality before mastectomy found that less than half of the participants had adequate knowledge of BR procedures and risks, with only 43% making a high-quality decision regarding BR [19]. A systematic review of 30 studies found 80% of women lacked information about both the possibility of BR and the range of BR options [20]. Although BR is associated with higher quality of life [8], many having BR experience dissatisfaction and difficulties. Dissatisfaction has been associated with unmet expectations regarding the physical appearance of the reconstructed breast, including lack of symmetry and different shape or feel and discrepancies between their expectations and their experiences of recovery [21–25]. A lack of realistic information may contribute to this [26].

Despite work showing that many Australian women undergoing mastectomy for breast cancer feel they lack information about BR, only a relatively small number of studies have examined their BR information needs, with much of this work focusing on the needs of specific groups of women, particularly those from regional or non-English-speaking backgrounds [17, 27]. Through qualitative analysis of free-text comments to an open-ended question asking what could be changed to improve BR experiences from large sample of women across Australia, this study aims to understand information needs and gaps relating to BR. As there are no mandates regarding the provision of BR information in Australia, understanding women’s perception of BR information gaps can assist in the development of strategies to meet these needs.

## Method

### Design

This study involves a secondary analysis of data from a cross-sectional survey undertaken by the Breast Cancer Network Australia (BCNA). A qualitative analysis of free-text responses was undertaken with themes derived from a phenomenological perspective [28]. Ethics approval was provided by DUHREC (2021-162). Where applicable reporting follows COREQ guidelines [29].

### Survey process

The survey was undertaken by BCNA and was open between April 10 and April 30, 2021. An invitation to participate in an online survey about BR experiences was emailed to 43,122 BCNA members who met the following inclusion criteria: diagnosis of non-invasive (Ductal Carcinoma in Situ or Lobular Carcinoma in Situ) or invasive breast cancer at any stage or at risk of breast cancer due to genetics/family history. People who had completed, were still having, and were undecided or decided against BR could complete the survey. A reminder email was sent to those not opening the invitation email.

The survey consisted of 55 questions, covering diagnosis, reconstruction status, BR type, health system treated in, costs, waiting times, satisfaction with outcomes and decisions, and factors influencing those undecided or had decided not to have BR (see Appendix 1 for more details on survey content). This study focuses on responses to the open-ended question: ‘Thinking about women who may experience a breast reconstruction in the future, is there anything you think needs to change so that they have a better experience?’. Demographics (age, state of residence, and postcode), breast cancer diagnosis, treatment, and BR experiences were also collected. Postcode information was used to infer residential location and socio-economic positions using the Australian Statistical Geography Standard (ASGS) [30] and the Socio-economic indexes for areas (SEIFA) [31] respectively.

### Data analysis

The free-text comments underwent thematic analysis, using an inductive approach [32], with all coding conducted in QSR NVivo 12. Coding procedures were adapted from those used for thematic analysis of written comments in the United Kingdom’s cancer patients’ experience of care surveys [33–35]. One researcher (KW) undertook a deep reading of all responses and developed potential codes noting recurring words, phrases, and meaning of comments. A second researcher (VW) familiarised herself with 50% of responses and developed potential codes. The two researchers compared codes, discussed similarities and differences, and agreed on a coding frame, which was then applied to all coded responses. KW used this coding frame for the remaining responses. During analyses, KW and VW had regular discussions regarding data and codes with VW reviewing random samples of coding. Adjustments to the coding frame were made when necessary. KW and VW met to discuss themes and subthemes emerging from the codes with these subsequently reviewed and refined. The research was undertaken as part of KW’s fourth-year psychology research project supervised by VW. VW has over 25 years of cancer research experience, including qualitative and quantitative research methods, and provided KW with training and guidance. Both KW and VW were female researchers with no relationships with survey participants and no experience of breast cancer and BR. KW kept reflexive memos to document thoughts and feelings generated when reading the comments.

Descriptive statistics described respondents in terms of their sociodemographic and oncology characteristics with chi-square tests used to examine whether there were differences in those respondents providing free-text comments and those not commenting.

## Results

Of 3384 completed surveys, 2077 respondents (61%) provided a comment to our question. Of respondents, 936 (28%) had decided against BR, 428 (13%) were still deciding, and 1985 (59%) had decided to have BR, including 1364 (40%) who had completed BR, 326 (10%) in the process of having BR, and 295 (9%) waiting for BR (see Table 1). Women providing a response were more likely to have completed their BR (48% vs 29%, p < 0.001), reside in a major city (p < 0.01), be aged in their 50s (p < 0.01), and reside in a high socio-economic area (p < 0.01) than those not providing a response (Table 1). Women not commenting were more likely to be satisfied with their BR outcome (88.4%) than those commenting (78.0%) (p < 0.001) (Table 1).

**Table 1:** Sociodemographic and satisfaction characteristics

Characteristics	Respondents who did not leave a comment		Respondents providing a comment	
	Number of respondents	Percentage (%)	Number of respondents	Percentage (%)
Total (N = 3384)	1307	39	2077	61
Age (n = 3217)	(n = 1140)		(n = 2073)	
18–39	70	6.1	130	6.2
40–59	577	50.9	1154	55.5
60–69	332	29.1	572	27.5
70 +	158	13.8	217	10.5
Residential location (n = 3110)	(n = 1088)		(n = 2022)	
Major city	743	68.3	1457	72.1
Inner regional	240	22.1	380	18.8
Outer regional	88	8.1	168	8.3
Remote	17	1.6	17	0.8
Socio-economic position (n = 3119)	(n = 1093)		(n = 2026)	
0–40% (most disadvantage)	319	29.2	481	23.7
41–80%	465	42.5	832	41.1
80–100% (least disadvantage)	309	28.3	713	35.2
Breast reconstruction decision status (n = 3349)	(n = 1272)		(n = 2077)	
Still deciding	206	16.2	222	10.7
Waiting group	104	82	191	9.2
In the process	91	7.2	235	11.3
BR completed	372	29.2	992	47.8
Decided not to have BR	499	39.2	437	21.0
Satisfaction with BR decision (n = 1625)	(n = 405)		(n = 1220)	
Very unsatisfied	2	0.5	31	20.5
Unsatisfied	10	2.5	58	4.8
Neither	15	3.7	87	7.1
Satisfied	145	35.8	373	30.6
Very satisfied	233	57.5	671	55.0
Satisfaction with BR outcome (n = 1321)	(n = 329)		(n = 992)	
Very unsatisfied	4	1.2	37	3.7
Unsatisfied	10	3.0	87	8.8
Not sure	24	7.3	95	9.6
Satisfied	133	40.4	329	33.2
Very satisfied	158	48.0	444	44.8

**Fig. 1:**
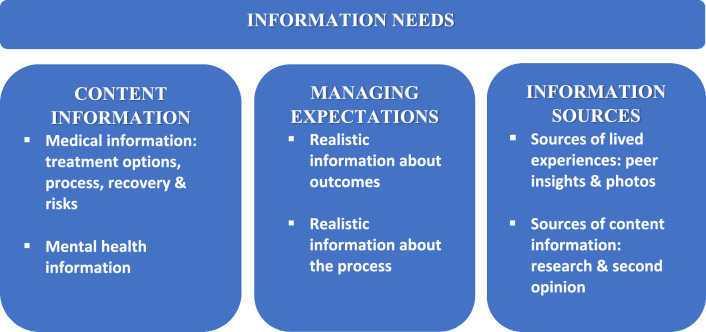
Themes and subthemes identified from free-text comments

Approximately, 60% of comments related to the type and sources of information needed. Three themes emerged: (1) content of information, (2) managing expectations, and (3) information sources, each associated with subthemes (see Fig. 1). The importance of BR information in managing emotions including reducing fears and anxiety was evident across the themes. Exemplar quotes supporting each theme and subtheme are provided in Table 2.

**Table 2:** Examples of written comments supporting each theme

Themes and subthemes	Illustrative quotes
Content information	
Medical information	
Treatment options (n = 164)	‘More information on the types of reconstruction available as well as the implants available. The positive and negative aspects need to be explained so that you can make an informed choice’
	‘Options need to be made available to them all. Not just what suits the specialist. If the specialist is unable to offer a specific outcome but knows it’s available, it should be discussed.’
	‘I think it should be discussed at diagnosis. I read about immediate reconstructions, but it was never something that was even discussed or offered as an option, if everything could have been done together, I may have been more inclined to do it.’
	‘I think that the option of flat closure needs to be spoken about more as a totally viable and acceptable ‘reconstruction’ option, and not just remaking breast mounds in some way.’
	‘More information about ALL the options including the option to go flat and be comfortable with that. I found the push/encouragement for reconstruction quite surprising—only through my own research did I discover that going flat for aesthetics as well as eliminating any potential risk of return was a real option.’
Process and recovery (n = 73)	‘For surgery to be clearly explained as to the steps, wait times, number of surgeries, likely recovery times’
	‘Step the patient through the process. .... tell [them] what will happen or could happen. It’s important to know exactly what is ahead, even if the questions haven’t been asked by the patient’
	‘To be kept informed about the length of the waiting time and not left wondering.’
	‘I think the potential costs need to be outlined early and without the need to have to ask for this.’
	‘More information about what happens after procedures and handy hints of what helps in recovery after surgery and what to bring to hospital
Side effects and risks (n = 85)	‘Pain in the tummy with reconstruction from your own tissue. If I had known it beforehand, I don’t think I would have agreed to go through it. Pain is not an issue for surgeons, and they play it down.’
	‘Maybe explaining more about how your body feels after reconstruction and the aches and pain you might have. The focus seems to be just on the surgery and the immediate recovery not the time after that.’
	‘No one said I would have no real feeling in my breast or that my abdomen will feel like it’s been laminated.’
	‘Better information about lymphoedema prevention and complications, such as post op infection & cording.’
Mental health information	
Psychological impact & support (n = 94)	‘Better preparation and support for psychological trauma experience from the point of diagnosis, through treatments and recovery. I have had a positive reconstruction but still grieve the loss of my breasts. I don't feel like I have breasts anymore, don't want to look in mirror or have anyone look at me. You can't trick your psyche in believing you are still a whole. No one prepared me for the loneliness having cancer can bring....even years after....It is a very personal trauma’
	‘More counselling needs to be provided before and after having a mastectomy and reconstruction! It doesn't instantly replace what you had before!’
Managing expectations	
Realistic information about outcomes (n = 60)	‘Surgeons need to be realistic about possibility of non-successful end results.’
	‘I do believe women should be made more aware that the breasts will not look the same. I had my normal breast reduced to at least be the same size. It is not. I look lop sided.’
	‘When something is going wrong with the reconstruction, being honest with the patient about what that means in terms of cosmetic outcomes—kept being told that things could be fixed, and they could not be fixed.’
Realistic information about the process (n = 61)	‘I was told it was a "big recovery" but maybe more specific information about what to expect physically post-op, i.e. the months of feeling like my stomach was on “the rack” being stretched, and the recovery time for me was 3 months not the 3- 6 weeks the plastic surgeon talked about.’
	‘More information about possible side effects to prepare mentally because numbness was a surprise to me.’
	‘Being fully informed on the process/steps, including a very realistic explanation as to what recovery from that type of surgery entails.’
Information sources	
Sources of lived experiences	
Peer insights (n = 86)	‘I had no communication with anybody about it aside from the surgeon! I would have loved to have spoken to a person who had experienced the same path I was going down plus I wanted to see what it looked like in reality if that was possible.’
	‘It would be helpful to have more access to photographs and real women’s experiences at the time of surgeon appointments. My surgeons have been fantastic and have done a wonderful job minimising my need for reconstruction, but I have felt quite overwhelmed sometimes not knowing what to expect my body to look like at different points throughout my treatment. Real world images rather than cartoons/medical sketches would have helped reduce my anxiety before surgery.’
Photos (n = 43)	‘I’d really like to see some before and after photographs, not just the great and terrible outcomes, but a selection of typical results, so that I can get a realistic understanding of the likely outcomes.’
	‘I think being shown pictures of women who have had nipple reconstruction would help more with that decision as you really aren’t sure what you’ll end up with by explanation only.’
Sources of content information	
Research (n = 60)	‘Research, research, and research again. You can never have too much information.’
	‘I had to do my own research if I wanted something other than what my surgeon offered.’
Second opinion ( n = 22)	‘They need to be told to consider a second opinion. The process is initially very quick, a lot of information, and can be quite daunting to think you have a choice when faced with so many experts. If I had not had the impetus to get a second opinion, I would not have found my surgeon and not had the very positive outcome that I had. I was at first presented with only the option of a complete removal of my breast, including nipple, and then a second process of expander and implant and eventual nipple reconstruct. Once seeking a second opinion I found I had a much different and better option.’

### Content of information

The largest theme encompassed the content of information that women wanted and was divided into two subthemes: (1) medical information, reflecting a need to be receive information regarding treatment options, process and risks involved, and (2) information needs regarding the psychological impact of BR. In both subthemes, there was a strong preference for information to be accurate and reflect negative as well as positive experiences (Table 2).

#### Medical information: treatment options, process, recovery, and risks

Women expressed the need for more comprehensive information on all reconstruction options. Most women wrote about their need to have clear explanations on the pros and cons for each option, with many describing the information they received as incomplete, rushed, or targeted towards procedures performed by their specialist. Some women reported a total absence of discussion around BR, which left them feeling disempowered and unprepared to make BR decisions. Comments suggested the lack of discussion was partly related to the breast surgeon not performing BR and not referring to others or assumptions around the woman being too old, overweight, or unable to afford BR. Some respondents discussed the need for information on the pros and cons of aesthetic flat closure, referred to as ‘going flat’, and to present it as a valid option. Women providing these comments described having to pressure their specialist to get more information on aesthetic flat closure whilst feeling pressured to have BR. Women also raised the importance of providing information on BR options prior to the commencement of any treatment or surgery. Provision of information at this time enabled a choice of immediate or delayed reconstruction, consideration of the impact of different treatments on type of BR possible post-treatment, and time to think about options and plan accordingly.

Commonly, women reported a need for more information around the process of having BR, including types of surgical procedures, implant options, possible number of surgeries, timeframes, waiting times, and cost. Having accurate information about these issues was commonly suggested as a way to reduce anxiety around undergoing BR. Waiting times were a source of dissatisfaction, with the majority of those treated in public hospitals reporting negative experiences. Women expressed the need to be kept informed about waiting times, delays, and surgery rearrangements. Whilst most comments regarding costs focused on excessive or prohibitive expenses in private hospitals, some expressed the need for clear upfront information about costs.

Many reported a need to be informed about the recovery process, including pain and discomfort, wound care, movement restrictions, and recovery times. Deficiencies in practical information about long-term recovery left women feeling overwhelmed and unsupported, when their recovery time was longer than expected. Coupled with this, was a need for information about possible side effects from surgery, including pain, loss of sensation in the reconstructed breast, numbness, scarring, and impact on physical abilities. Many women also expressed the need for clearer information about risk of infection, lymphoedema, implant encapsulation, and extra surgeries (see Table 2 for exemplar comments).

#### Mental health information

This theme related to managing and maintaining mental health. Women described BR as ‘emotionally challenging’, ‘overwhelming’, and ‘highly stressful’, particularly when needing to make quick decisions, grieving the loss of breasts, accepting extensive body changes, or endlessly waiting for surgeries. Some women gave examples of how BR had negatively impacted their self-esteem or sexual well-being. Subsequently, many women reported the need to be given more information about BR’s potential psychological impact and counselling services that might be used before, during, and after BR.

### Managing expectations

The second theme related to information needed to help manage expectations around BR. Comments described how the absence of this type of information meant many experienced a gap between BR expectations and their experiences. This gap was associated with dissatisfaction with BR outcomes and decision regret. Two subthemes were identified reflecting the need for realistic information in two areas: (1) physical outcomes and (2) the BR process, including recovery (see Table 2 for exemplar comments for these subthemes).

#### Realistic information about outcomes

Respondents commonly reported a need for more ‘honest’ information about the potential size, shape, asymmetry, scarring and loss of sensation in reconstructed breasts, and how different the breast could feel from their own. Women wanted information that provided them with more realistic expectations about outcomes, including how the result may not be flawless.

#### Realistic information about the process

Some women reported that having realistic expectations about the BR process helped them to prepare mentally for the ‘struggles ahead’. Many others regretted a lack of preparedness and reported being surprised by the many unexpected struggles, including multiple surgeries, longer and more painful recovery periods than presented, or unanticipated lack of mobility and complications. This information enabled women to develop realistic expectations regarding recovery and outcomes and was crucial in allowing women to cope with and manage any difficulties and complications that may arise.

### Information sources

The third theme reflected the information sources that could provide women with the realistic information they sought. Two subthemes were identified: (1) sources of lived experiences and (2) sources of content information.

#### Sources of lived experiences: peer insights and photos

This subtheme highlighted how peer insights were considered a crucial source of information for women at the start of their BR journey. It reflected the importance placed on receiving realistic information, with peers seen as being able to discuss their lived experience of having or not having BR. These interactions contributed to reduce fear, helped women feel more informed, and helped decision-making. Many regretted not having access to such discussions. Similarly, many expressed the need to be shown additional and more realistic before and after photos showing outcomes with different breast sizes and shapes.

#### Sources of content information: research and second opinion

The second subtheme focused on the role women could have in sourcing information. Many commented on the importance of taking time to research and educate themselves about both BR options and available surgeons and to ask as many questions as necessary. Comments noted the importance of being able to get a second opinion with this seen as an important source of information associated with receiving a wider range of BR options and communication styles to choose from. Those who could adopt these roles felt more able to make decisions that were appropriate for them.

## Discussion

Despite being included in guidelines for delivery of optimal breast cancer care, there is no formal mandate for women treated by mastectomy for breast cancer in Australia to receive information about BR. In this environment, it is important to understand the information needs and gaps women experience. The study identified three themes relating to the content of information needed, managing expectations and sources of BR information. Our study’s findings suggest that women need information that is comprehensive, realistic regarding possible outcomes and experiences, and addresses the potential psychological impact of BR.

A key finding from our study was the need for more comprehensive and timely information about multiple aspects of BR, including options, procedures, outcomes, and recovery. Many women were not informed about the full range of BR options, with many only having BR discussions post-mastectomy. These findings are similar to previous research from Australia [18] and the US prior to 2016 [19] that found women lack timely information regarding possibility of immediate reconstruction, with Australian research also finding that women are frequently only informed about BR procedures performed at their local health service [27]. Whilst position statements developed by peak bodies (e.g. Australian Society of Plastic Surgeons, Breast Surgeons of Australia and New Zealand [36]) aim to create consistency of information provision, these are only guidelines and currently there is no standardised procedure for discussing BR with women in Australia. Our results show the impact of this, with the many information gaps reported.

This study found a need for more realistic information particularly in relation to possible physical outcomes, with many respondents indicating a lack of preparedness for the look, shape and feel of their reconstructed breast. These findings are similar to research from the US, New Zealand [26], Canada [37], and the UK [38], suggesting the universality of this need. Evidence suggests that decision regret is reduced when women are satisfied with information about likely BR outcomes and can set their expectations accordingly [39]. These findings suggest the need to develop strategies for effective communication regarding physical aspects of having breast implants or autologous reconstruction, which acknowledges that outcomes may differ between women. Providing a greater range of information that reflects women’s lived experiences of BR may help manage expectations and lower decision regret.

More realistic information regarding the BR process, recovery time and experiences, and possible physical response was a key information need. In the absence of this information, many women felt unprepared for the pain, side effects, additional surgeries, complications, and lengthy recovery period they experienced. The lack of realistic information about recovery prevented women from anticipating potential difficulties and reduced capacity to cope with the BR process. Others have also reported a lack of preparedness around the side effects experienced during BR [22, 25]. In their meta-synthesis, Car et al. [25] reported that many women felt that the information they received did not accurately reflect the realities of the recovery process. Similar to others [22], our study suggests the mismatch between BR experiences and expectations was associated with BR dissatisfaction. Greater emphasis needs to be placed on providing realistic information about challenges throughout the process, to assist women to plan for possible impact on work or caring responsibilities.

Our results highlight how emotionally challenging BR is and the impact it can have on women’s emotional states, body image, self-esteem, and sexual well-being. Women expressed the need for more information on the potential psychological impact of BR along with information on psychological services that might help manage this impact. Whilst other work has suggested the emotional impact of mastectomy and BR [26, 40], few have explored these issues in detail. Our findings suggest the need for health professionals to acknowledge and address the potential psychological impacts of both having BR and waiting for BR. The delay in BR access that some women in Australia experience also suggests that community organisations and breast cancer-specific support services need to be alert to the emotional needs of this group and work towards identifying services they can use whilst waiting for and/or during BR.

Lived experiences and peer insights appeared to be a needed source of BR information as it provides opportunities to hear testimonies, ask unanswered questions, and see or feel reconstructed breasts. Similar to others [40], our findings suggest that peer exchanges enabled women to gain a realistic appreciation of likely BR outcomes, the process of having BR, and an understanding of the emotional impact of undergoing BR. Realistic post-reconstruction photos were also considered beneficial. Research regarding the impact of peers in the BR process has focused mostly on the role of peers in the context of psychological support post-BR [28, 41, 42]. Our finding extends the literature and suggests that exchanging with other women regarding their BR experiences is a crucial source of information for women considering BR. Further, work is needed to investigate the best ways to connect peers with women who are at the beginning of their BR journey, which is complicated by the limited timeframe for decision-making.

A significant minority of respondents in our study wanted more information about the possibility of having an aesthetic flat closure and, importantly, for surgeons to present aesthetic flat closure as a legitimate option. Some women were confronted with a lack of acceptance and reluctance from surgeons to discuss aesthetic flat closure and felt pressured to have a reconstruction. Work from the US has found that women perceive a low level of surgeon support if they decide to go flat and this is often the strongest predictor for low satisfaction with surgical outcomes [43, 44]. This study extends the literature by highlighting that some women have information needs around non-reconstruction options, which are often not supported by surgeons. Further research is required to investigate women’s and surgeons’ attitudes towards the option of going flat after mastectomy for breast cancer.

Decision aids may be one strategy of providing women with the information needed to make decisions regarding BR. Whilst an Australian decision aid exists [45, 46], there is limited information regarding its use. As decision aids for BR have been shown to reduce decision regret and improve decision satisfaction [46, 47], work is needed to develop strategies to ensure women access these resources.

Our study had limitations. The free-text comments were brief and in response to a specific question, limiting their qualitative nature. However, this was compensated by the large number of comments which enabled data saturation and by the fact that the sample included women from across Australia and women with positive and negative BR experiences. As some women may opt to have BR several years after their mastectomy, we included women who had their breast cancer diagnosed up to 10 years previously to capture these experiences. As some women had their BR more than five years ago, some comments might not reflect current information provision. Whilst our findings regarding the BR information needs and gaps may be most relevant to countries without mandates for the provision of BR information, we believe our findings regarding the content of information particularly information relating to the management of the emotional impact of BR, is relevant everywhere.

## Conclusion

Free-text analysis from a large sample highlighted current gaps in the content and format of information Australian women receive about BR. Whilst our study supports previous findings in highlighting gaps in information relating to BR options, potential outcomes and side effects, it also highlighted the need for more information about the recovery process and the emotional impact of BR. Realistic information was valued and helped to manage expectations and inform women about BR potential psychological impact. Our study underlined the importance of women considering BR to hear the lived experiences of peers as well as having access to realistic pre- and post-surgery photos to better inform and prepare women for the process of BR if this was their decision. Future studies should investigate ways to provide realistic information about possible positive and negative outcomes associated with BR process, including recovery.

## Data Availability

The data used for this study are not publicly available due to privacy and ethical restrictions. Enquiries relating to the data should be directed to the corresponding author in the first instance.

## Appendix

Description of Survey tool.

The current study was based on analyses of free-text responses to an open-ended question at the end of an online survey that focused on Australian women with breast cancer experiences of Breast Reconstruction (BR). In this Appendix, we provide more detail about the survey tool.

The survey was designed to gather information about the BR experiences of those at different stages of BR: (1) completed BR; (2) currently in the process of BR; (3) planning to have BR; (4) undecided about BR; and (5) decided against BR. The questionnaire was structured to direct people in these different BR stages to relevant questions.

All participants completed the first section of the survey assessing

- Stage of breast cancer at diagnosis or most recent diagnosis,
- Time since diagnosis,
- Treatment (type of surgery, radiotherapy, chemotherapy, hormone blocking therapy, targeted therapy), and
- Breast reconstruction status (completed, currently having, planned, undecided, decided against).

Respondents who had completed or were currently having BR were asked questions relating to

- Type of reconstruction (implant, autologous),
- Nipple reconstruction,
- Timing of the reconstruction (e.g. immediate, delayed),
- Health system for reconstruction (private or public system),
- Experience of delays and communication from health services about delays,
- Distance travelled for surgery,
- Access to state-based travel assistance schemes,
- Out-of-pocket costs if treated in the private system,
- Number of surgeries, and
- Satisfaction with the decision to have reconstruction,

Those who had completed BR were also asked for their satisfaction with the outcome.

Those still undergoing BR were asked if COVID-19 pandemic had impacted on their treatment.

Women planning to have breast reconstruction were asked

- If they were on a waiting list and if so the length of time waiting,
- If they were told how long the wait may be,
- Health service where BR would take place (public or private),
- Expected costs if BR was in the private system,
- Type of reconstruction they were planning to have, and
- Timing of the surgery.

Those undecided and those deciding not to have reconstruction were asked to select influences on their decision from a list of 10 items that included costs, distance, importance of breast reconstruction to the woman and her family, waiting times, time needed to recover, and lack of information about the procedure.

Those indicating that distance, costs, or waiting times influenced their decisions were asked to indicate what these items would have been for them if they were to have reconstruction.

All respondents were asked to complete a number of demographic questions including

- Age (in eight 10-year age groups with 80 + years the highest),
- State of residence,
- First nations person status,
- Language spoken at home, and
- Residential postcode.
